# The frontal and posterior cortical areas involved in the non-spatial visual allocation of attention in the human brain: a functional neuroimaging study

**DOI:** 10.3389/fnins.2024.1472114

**Published:** 2025-01-22

**Authors:** Kristina Drudik, Veronika Zlatkina, Elise B. Barbeau, Michael Petrides

**Affiliations:** ^1^Department of Neurology and Neurosurgery, Montreal Neurological Institute, McGill University, Montreal, QC, Canada; ^2^Department of Psychology, McGill University, Montreal, QC, Canada

**Keywords:** allocation of attention, non-spatial, fMRI, dorsal attention network, cytoarchitectonic area 8A, if/then conditional rules

## Abstract

Previous functional neuroimaging studies had demonstrated the involvement of cytoarchitectonic area 8Av of the prefrontal cortex in the cognitive allocation of attention to spatial stimuli. The present functional magnetic resonance imaging (fMRI) study examined brain activity related to the allocation of attention to *non-spatial* visual stimuli, i.e., stimuli that are defined by their perceptual features and are independent of their location. The study established (a) the involvement of area 8Av in the allocation of attention to non-spatial stimuli in the environment and (b) the areas co-activated with area 8Av across the entire cortex so that the complete functional cortical network could be defined. Finally, based on individual subject analysis, the functional activity in area 8Av was related to specific sulci in the caudal middle frontal gyrus. The novel information provided by the current fMRI study significantly advances our understanding of the role of area 8Av in the selective allocation of attention to stimuli in the environment.

## 1 Introduction

**FIGURE 1:**
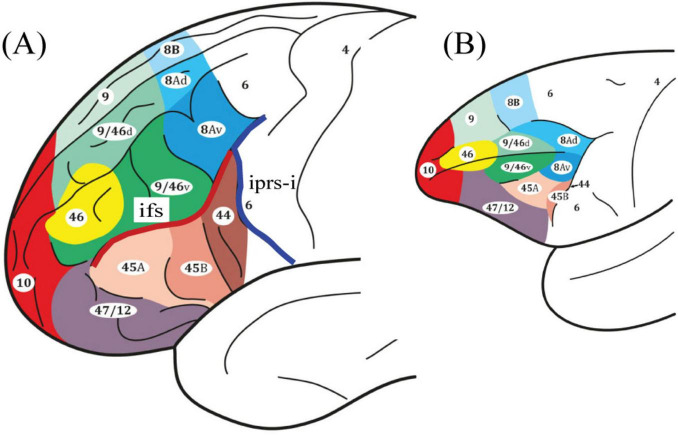
Schematic illustrations of the lateral surface of the cerebral cortex. Comparative cytoarchitectonic map of the frontal lobe in panel **(A)** the human and **(B)** the macaque monkey brains (Petrides and Pandya, 1999; Petrides and Pandya, 2002). Note that area 8A is denoted in blue and the ventral boundary is found at the intersection of the inferior frontal sulcus (ifs) in red and the inferior ramus of the inferior precentral sulcus (iprs-i) in dark blue. All figures reproduced with permission.

The prefrontal cortex is a large expanse of cortical areas that underlie executive processing, namely the higher order control processes that regulate activity in posterior cortical areas (Luria, 1973; Petrides, 2005; Shallice and Burgess, 1996; Figures 1A, B). Research in patients has demonstrated an impairment in the selective allocation of attention to stimuli in our distraction-filled environment after prefrontal cortical damage (Petrides, 1985a; Petrides, 1990). In macaque monkeys, lesions restricted to the periarcuate region, where area 8Av lies, produced a comparable impairment in the selective allocation of attention to exteroceptive stimuli (Petrides, 1985b; Petrides, 1987). In these experiments, on a trial-by-trial basis, subjects were required to select the appropriate target stimulus from a set of competing stimuli, based on conditional rules: if instruction cue A is presented, attend to and select visual target stimulus X, but if instruction cue B is presented, attend to and select visual target stimulus Y. Furthermore, functional neuroimaging in healthy human

participants demonstrated increased activity in area 8Av on the middle frontal gyrus (MFG) (see Figure 1A) when allocating attention to particular locations based on conditional rules (Germann and Petrides, 2020a; Germann and Petrides, 2020b). It is important to note that lesion studies in patients and macaque monkeys had demonstrated that the spatial element was not a necessary condition for the executive control process of the selective allocation of attention (Petrides, 1985a; Petrides, 1985b; Petrides, 1990). Thus, the question that now emerges is whether the activation of area 8Av in healthy human subjects that was observed in functional neuroimaging studies applies only to the selective allocation of attention to the spatial aspect of sensory stimuli, i.e., their location, or also to non-spatial stimuli (e.g., objects, faces, designs, etc., regardless of their location).

**FIGURE 2:**
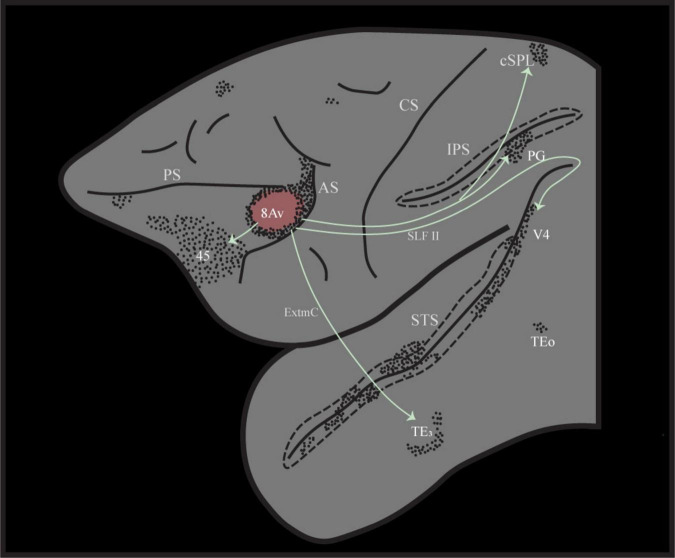
Illustration of the distinct cortico-cortical connectivity pattern following retrograde tracer injection into area 8Av in the macaque monkey, case 3 from (Petrides and Pandya, 1999). Note that area 8Av is connected with ventrolateral prefrontal area 45 involved in selective memory retrieval, ventral temporal cortex for the perceptual processing of visual stimuli, as well as intraparietal sulcal cortex and adjacent posterior inferior parietal cortex (area PG) that provide the spatial context within which perceptual stimuli are embedded. AS, arcuate sulcus; CS, central sulcus; cSPL, caudal superior parietal lobule; ExtmC, temporo-frontal extreme capsule fasciculus; IPS, intraparietal sulcus; PS, principal sulcus; SLF II, superior longitudinal fasciculus II; STS, superior temporal sulcus.

The precise examination of cortico-cortical connectivity in non-human primate brains has demonstrated specific anatomical circuits that are the gold-standard for the accurate interpretation of cortical functional networks (Petrides and Pandya, 1999; Petrides and Pandya, 2002). Area 8Av was shown to be connected with ventrally adjoining prefrontal area 45, which is involved in the selective retrieval of previously acquired information (Kostopoulos and Petrides, 2003; Kostopoulos and Petrides, 2008), the intraparietal sulcus and the adjacent area PG in the posterior inferior parietal lobule, which is involved in the processing of the spatial context within which stimuli are embedded (Astafiev et al., 2003; Mishkin et al., 1983; Petrides and Iversen, 1979), and the occipito-temporal region involved in the perceptual processing and recognition of visual stimuli (Allison et al., 1999; Astafiev et al., 2003; Kanwisher et al., 1997; Mishkin et al., 1983) [Figure 2; Case 3 in (Petrides and Pandya, 1999)].

Based on the information provided above, the objectives of the present functional magnetic resonance imaging (fMRI) study were, first, to examine whether area 8Av is involved in the selective allocation of attention to visual stimuli in the environment that are defined by their perceptual features and are *independent of their location*. Second, based on macaque connectivity studies, we hypothesized that area 8Av in the human brain would be co-activated with other specific cortical areas and, thus, reveal the functional network for the high-level control process of the selective allocation of attention to stimuli in the environment. Specifically, we examined whether, during the conditional allocation of attention to non-spatial stimuli in the environment, there was co-activation of area 8Av with adjacent area 45 that is involved in selective memory retrieval, with premotor frontal eye field areas that organize eye movements, the posterior intraparietal cortical region that is involved in the perception of stimuli within space, as well as with the occipito-temporal region involved in the perceptual processing of visual stimuli.

## 2 Materials and methods

### 2.1 Subjects

Twelve healthy human subjects (six female) participated in this fMRI study. The ages of these participants ranged from 20 to 31 years (mean age 25 years) and they were all right-handed. The subjects provided informed written consent to participate in the study according to the institutional guidelines established by the Research Ethics Committee of the Montreal Neurological Institute (MNI).

### 2.2 Experimental design

**FIGURE 3:**
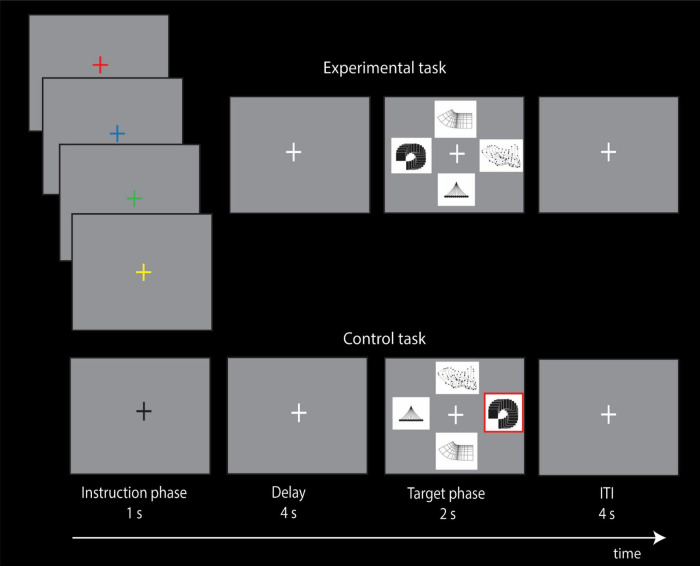
Experimental design of the non-spatial visual conditional attention task and the control task. In the instruction phase of the four experimental conditional trials, the subjects are shown an instruction cue (red, blue, green, or yellow colored cross) that instructs the allocation of attention to the appropriate target stimulus (abstract visual design) based on previously learned conditional rules. In the instruction phase of the control trial, a black cross is shown that does not instruct the allocation of attention to any visual perceptual stimulus. The subjects had learned before the scanning session that each one of the four visual instruction cues instructed the allocation of attention to a particular one of the four visual target stimuli (abstract visual designs), i.e., if red fixation cross, attend cognitively to abstract target stimulus X, but if blue fixation cross, attend cognitively to abstract target stimulus Y, etc. Note that during the 1 s presentation of the colored instruction cue, the subject is *cognitively* focusing attention on the particular abstract visual design that is associated with the instruction cue because the design stimuli are not on the screen. The design stimuli appear later, after a delay of 4 s, during the target selection phase of the trial and the subject presses the appropriate button to indicate the target to which they had allocated attention when the colored instruction cue was presented earlier in the instruction phase of the trial. Note that, during the target phase of the trial, the location of the abstract designs varies randomly, so that there is no relation of any spatial location with any of the four abstract visual designs. In the control trials, a black fixation cross was presented during the instruction phase that did not instruct the allocation of attention to any of the abstract visual designs. In the control trial, the subjects had been trained to select, during the target phase of the trial, the abstract visual design that was outlined in red, and the design outlined in red was randomly determined. Thus, the instruction cue during the control trials carried no information as to which of the four abstract visual designs should be attended to. Note also that, in the target phase of the trials in both conditions, all four targets were presented and changed their locations randomly on a trial-by-trial basis. Thus, participants could not rely on spatial information to make their cognitive selection in the instruction cue phase of the trial or during the target phase of the trial when they were selecting the non-spatial stimulus to which they had allocated attention based on the visual instruction cue. The intertrial interval (ITI) was 4 s.

The present event-related fMRI study was designed to examine brain activity during the allocation of attention to *non-spatial* visual stimuli based on if/then conditional rules that had been learned prior to scanning. Toward that end, the subjects performed two tasks: a non-spatial visual conditional experimental task and a control task. In the experimental trials, the subjects were required to focus attention on a particular visual stimulus based on conditional instruction cues, i.e., if instruction cue A is presented, cognitively allocate attention to target stimulus X, but if instruction cue B is presented, allocate attention to target stimulus Y, etc. In the control trials, a neutral instruction cue was presented that did not indicate the allocation of attention to any one of the target stimuli and, thus, did not involve the instructed cognitive allocation of selective attention (Figure 3).

In the experimental condition, the subjects had learned prior to scanning that each one of four visual instruction cues (red, blue, green, or yellow colored fixation crosses) instructed the allocation of attention to one of the four visual target stimuli (abstract visual designs). In other words, the presentation of a visual instruction cue (e.g., green colored fixation cross) indicated the cognitive allocation of attention to the particular abstract design that was associated with it. By contrast, in the control condition, during the instruction phase of the trials, a black fixation cross was presented that did not indicate the allocation of attention to any one of the four abstract designs. The instruction cue in both the experimental and control trials was followed by a 4 s delay and then the subjects were presented with the four abstract designs during the target phase of the trial and had to select the visual abstract design to which attention had been cognitively allocated when the instruction cue was presented (Figure 3). The location of the visual abstract stimuli varied randomly during the target phase of the trials and, thus, there was never any relation between the visual abstract designs and particular locations. In other words, at the instruction phase of the experimental trials, the particular color of the fixation cross instructed the cognitive allocation of attention to a particular abstract visual design that had no link to any particular location. In the target phase of the control trials, one of the visual stimuli was outlined in red and the subject had to select that stimulus (Figure 3). Thus, during the control trials, the selection of the target stimulus was determined externally (i.e., outlined in red), by contrast to the experimental trials in which the target stimulus to be selected was the particular abstract design to which attention was allocated earlier during the instruction phase based on the color of the instruction cue. Thus, the comparison of brain activity during the instruction phase (duration of 1 s; see Figure 3) of the experimental trials with the instruction phase of the control trials would show activity related to the cognitive allocation of attention to specific *non-spatial* visual stimuli, i.e., the visual abstract designs that were not related to any location.

On the day prior to the fMRI scanning session, each subject had learned the four conditional associations through trial-and-error and had been instructed to maintain fixation on a cross that was present for the entire duration of each trial. The subjects practiced learning trials until they had reached a performance level of more than 90% accuracy on two consecutive sets of 32 trials. During the fMRI scanning session on the following day, each run consisted of both experimental and control trials that were presented in a pseudorandom order on successive trials, and each one of the conditional instruction cues (i.e., the four colored crosses) appeared four times mixed with an equal number of trials presenting the control cue (black fixation cross). Each trial started with the instruction phase, during which a colored fixation cross was presented for 1 s instructing the subjects to allocate *cognitively* attention to one of the visual abstract target stimuli based on the conditional rules that they had previously learned (Figure 3). Note that the target stimuli were not present on the screen at this instruction phase of the trial. The presentation of the instruction cues was random but balanced since each instruction cue was presented four times across each run. A delay of 4 s followed the instruction phase, and then the target phase (2 s) when all four abstract visual target stimuli were presented, and the subject had to select the target stimulus that had been indicated earlier by the instruction cue. For both experimental and control trials, there was a fixed intertrial interval of 4 s. The locations of the target stimuli changed randomly on a trial-by-trial basis and, therefore, the participants could not rely on spatial information to make their selection but were rather required to attend to and select the non-spatial visual stimulus indicated by the instruction cue based on the previously learned associations between the instruction cues and the abstract designs. In the target phase of each trial, the subjects pressed the correct one of four buttons on a response pad to indicate the current position of the target stimulus that was attended earlier based on the color of the instruction cue on that particular trial. The trial ended with a blank screen.

Note that only the experimental condition involved the instructed cognitive allocation of attention to non-spatial visual perceptual stimuli during the instruction phase of the trial since the black cross in the control trials did not instruct the allocation of attention to any one of the four visual abstract designs and, thus, a comparison of brain activity during the instruction phase of the experimental trials with the instruction phase of the control trials would demonstrate activity related to the covert cognitive allocation of attention.

### 2.3 MRI acquisition

Scanning was performed using a 3T Magnetom Prisma MRI Scanner (Siemens, Erlangen, Germany), with a 32-channel head coil at the McConnell Brain Imaging Center of the MNI. During the scanning session, an anatomical T1-weighted structural brain image was obtained with a gradient-echo MPRAGE sequence (192 slices, interleaved, voxel size = 1.0 mm^3^, TR = 2300 ms, TE = 2.96 ms, flip angle = 9^0^, slice thickness = 1 mm, FOV = 256 mm). Subsequently, six runs of 525 images each (48 oblique slices, voxel size = 3.0 mm^3^, slice thickness = 3.0 mm, TR = 800 ms, TE = 30 ms, flip angle = 60^0^) sensitive to the blood oxygenation level-dependent (BOLD) signal were acquired. Visual stimuli were presented through an LCD projector with a mirror system and the responses of the subjects were recorded using an MR-compatible optical response panel. Both stimulus presentation and the recording of the responses of the subjects were controlled with E-prime (E-prime 3.0; Psychology Software Tools Inc., Pittsburgh). Each of the six runs lasted approximately 6 min and consisted of 32 trials during which the experimental and control trials were presented in a pseudo-random order. The first trial onset in each run was synchronized with the scanner acquisition using a trigger signal generated by the scanner. Both behavioral and functional imaging data were acquired in all trials.

### 2.4 fMRI data preprocessing

Functional data were preprocessed and analyzed using Statistical Parametric Mapping software (SPM12; Wellcome Department of Cognitive Neurology, UCL, UK). Preprocessing was carried out using the standard steps: all images were realigned, unwarped, and fieldmaps were used for distortion correction. The scans of each participant were corrected for slice timing using the second slice as reference, co-registered to the T1 anatomical scans acquired in the same scanning session, and segmented into gray matter, white matter, and cerebrospinal fluid (CSF). Head motion correction was applied using a 6-parameter rigid-body alignment to model out potential non-linear head motion artifacts. Functional and morphological images were then spatially normalized into the MNI standard stereotaxic space using SPM’s default template, the ICBM152MNI space (Collins et al., 1994). Finally, the normalized functional images were smoothed using a 6 mm full-width half-maximum (FWHM) isotropic Gaussian kernel.

### 2.5 Statistical analysis

The event-related paradigm examined the difference in BOLD activity during the 1 s instruction phase of the experimental trials in comparison with the instruction phase of the control trials to determine the brain regions involved when allocating attention to non-spatial visual stimuli based on previously learned conditional associations. The onset of the conditions was the presentation of an instruction cue (green, red, yellow, or blue colored fixation crosses) that indicated the allocation of attention to a specific target stimulus (abstract visual design) in the experimental conditional task, or the presentation of a neutral cue (a black fixation cross) that did not indicate the allocation of attention to any target stimulus in the control condition (see Figure 3).

At the first level, the statistical analysis of the fMRI data was based on univariate linear modeling with correlated errors, using a design matrix including the experimental and control conditions, along with six movement parameters (three translation, three rotations), and a constant term included as covariates of no interest. A high-pass filter cutoff of 128s was used to remove low-frequency noise. Each trial was modeled with impulse regressors at the time of the presentation of the instruction stimulus. These regressors were then convolved with the canonical hemodynamic response function and entered into a general linear model (GLM) of each subject’s fMRI data. Sufficient sampling across the hemodynamic response function was acquired in this experiment because of the complete desynchronization of the trial onset time from the acquisition repetition time. This desynchronization was achieved with a repetition time of acquisition (TR, 0.8 s) and a total trial duration of approximately 11 s for both the experimental and control trials. At the second level, parameter estimates through a full factorial model were used to compare the two contrasts obtained for the experimental and control conditions for each participant at the group level.

The resulting *t*-statistic images were thresholded using Bonferroni correction and random field theory, which considers spatial correlation of the error (Worsley et al., 1996). Significance was assessed based on the spatial extent of consecutive voxels. At the second level, a whole-brain search was carried out, where a single voxel within an estimated gray matter volume of 600 cm^3^ covered by the slices was significant at a threshold of *t* = 4.58 (p*_corrected_* < 0.05) (Worsley et al., 1996; Worsley et al., 2004). At the first level and for single subject exploration, a directed Region of Interest (ROI) analysis was performed, where the estimated ROI included the MFG bilaterally, a region reported to have a volume of 9.63 and 10.21 cm^3^ in the left and right hemispheres, respectively (Goldberg et al., 2013). A predicted cluster of voxels with a *t*-value > 3 was significant (p*_*corrected*_* < 0.05) when its threshold extent was >376 mm^3^, corrected for multiple comparisons (Friston et al., 1995). Functional imaging data of individual subjects were superimposed on their respective anatomical volumes, all transformed into the standard MNI stereotaxic space, to examine the relationship between gyral and sulcal morphology and functional activity within cortical regions.

## 3 Results

The average success rate for all trials was 99.05% (range 93.75–100%), with a mean success rate for experimental trials of 98.26%, and for control trials of 99.83%. To isolate the functional activity associated with the cognitive allocation of attention to non-spatial visual stimuli (the abstract visual designs), we compared activity in the experimental trials with that in the control trials at the precise time that the instruction cue was presented, i.e., the 1 s instruction phase period. We hypothesized that, at the moment of the presentation of an instruction cue, the subjects would cognitively allocate their attention to the appropriate target stimulus based on the previously learned conditional rules. Furthermore, we examined functional activity in individual subjects by superimposing the *t-*statistical map computed for each individual subject’s functional activity (non-spatial visual attentional trials minus control trials) onto the subject’s respective anatomical MRI, aligned to the coordinates of the MNI standard stereotaxic space.

### 3.1 Frontal activations

**FIGURE 4:**
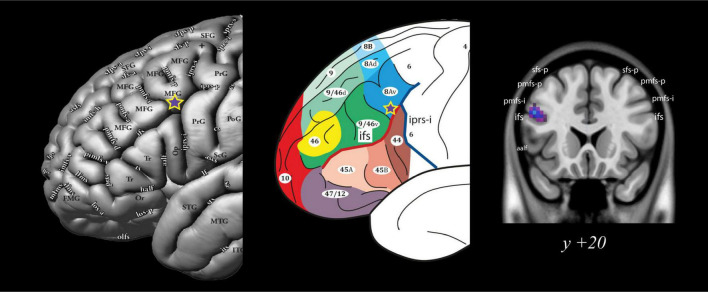
Group level analysis: Location of the activation peak in area 8Av during the conditional allocation of attention to non-spatial visual stimuli. The significant peak is located at the ventral boundary of the caudal middle frontal gyrus at the junction with the caudal inferior frontal sulcus (ifs), i.e., where area 8Av is located (Petrides and Pandya, 1999; Petrides, 2019). Note the purple star with the yellow outline denotes the significant functional activity peak obtained from the comparison of the instruction phase in the experimental non-spatial visual conditional attention task minus the instruction phase of the control task. See Petrides (2019) for additional abbreviations not found in the present text. All figures reproduced with permission. aalf, ascending anterior ramus of the lateral fissure; ifs, inferior frontal sulcus; iprs-i, inferior ramus of the inferior precentral sulcus; pmfs-i, intermediate ramus of the posterior middle frontal sulcus; pmfs-p, posterior ramus of the posterior middle frontal sulcus; sfs-p, posterior ramus of the superior frontal sulcus.

The group-level analysis demonstrated a significant activity cluster in the left hemisphere in the posterior MFG, where area 8Av is found (Petrides and Pandya, 1999) [MNI coordinate (*x, y, z*): = −48, 20, 29, *t* = 5.2, p_*FWE*_ = 0.0063] (see Figure 4 and Table 1). When functional activity was examined on a subject-by-subject basis, a significant increase in activity was observed consistently in the left posterior MFG (area 8Av) in ten out of the twelve subjects [average MNI coordinate (*x, y, z*): −43.1 ± 5.5 SD, 19.4 ± 3.9 SD, 33.9 ± 6.2 SD]), with an additional subject showing an activity cluster in the ROI in the left hemisphere that approached the standard level of significance (Table 2). A similar activation was seen in the right hemisphere, reaching significance in seven out of the twelve subjects [average MNI coordinate (*x, y, z*): 42.6 ± 5.1 SD, 20 ± 4.9 SD, 35.3 ± 7.8 SD]), with one subject showing an activity cluster in the ROI in the right hemisphere that approached significance (Table 2). Thus, in individual subjects, bilateral activation within the predetermined ROI was frequently observed, although it did not always reach the standard level of statistical significance. When examining the MRI volumes of the brain of each individual subject, the significant activity was consistently observed within the ventrocaudal MFG, above the inferior frontal sulcus (ifs), where area 8Av is located (Petrides and Pandya, 1999; Petrides, 2019) (see Figures 1, 4).

**TABLE 1:** Location of significant functional activity peaks across the entire brain from the group-level analysis during the fMRI experiment from the comparison of the instruction phase of the non-spatial conditional experimental trials minus the instruction phase of the control trials.

Location		MNI coordinate			
		*x*	*y*	*z*	*t-*value
Pars triangularis (anterior area 45)	LH	−48	32	−1	5.1*
Caudal middle frontal gyrus (area 8Av)	LH	−48	20	29	5.2*
Dorsal premotor saccade area (FEF, area 6)	LH	−29	−4	65	7.3*
Ventral premotor eye area (area 6)	LH	−48	5	33	6.1*
Supplementary frontal eye field (SEF, area 6)	LH	−3	−4	74	9.3*
Supplementary frontal eye field (SEF, area 6)	RH	3	−1	72	9.5*
Anterior cingulate eye field	LH	−2	17	33	9.3*
Precentral gyrus (area 4)	LH	−54	−10	43	5.9*
Precentral gyrus (area 6)	LH	−42	−19	62	6.5*
Fusiform gyrus	LH	−36	−55	−18	5.8*
Fusiform gyrus	RH	30	−49	−18	5.5*
Fusiform gyrus	LH	−52	−64	−16	6.2*
Fusiform gyrus	RH	33	−61	−19	4.0*
Intraparietal sulcal cortex	LH	−30	−58	59	6.1*
Intraparietal sulcal cortex	LH	−18	−73	51	5.2*
Posterior superior parietal lobule (SPL)	LH	−10	−70	59	5.7*
Intraparietal-Paraoccipital sulcal cortex	LH	−30	−85	26	4.4*

Maxima of regions showing an increase in BOLD signal (*P < 0.05, significant peaks). All coordinates are in standard Montreal Neurological Institute (MNI) stereotaxic space in both the left and right hemispheres. FEF, frontal eye field; LH, left hemisphere; RH, right hemisphere.

**TABLE 2:** Location of the functional activity peaks observed in the caudal middle frontal gyrus (area 8Av) in individual subjects from the comparison of the instruction phase of the non-spatial conditional experimental trials minus the instruction phase of the control trials.

	MNI coordinate			
	*x*	*y*	*z*	*t-*value
***Left hemisphere***				
Subject 1	−51	17	29	19.9*
Subject 2	−42	20	35	9.4*
Subject 3	−45	14	35	30.0*
Subject 4	−38	17	23	3.4
Subject 5	−33	23	32	7.9*
Subject 6	−39	26	47	23.2*
Subject 8	−39	20	32	8.7*
Subject 9	−48	23	35	12.9*
Subject 10	−42	20	23	5.7*
Subject 11	−42	17	38	6.1*
Subject 12	−50	14	33	14.0*
***Right hemisphere***				
Subject 1	39	14	26	12.7*
Subject 2	39	17	41	5.1*
Subject 3	51	20	35	7.9*
Subject 6	42	29	41	20.3*
Subject 8	42	17	23	11.2*
Subject 9	48	23	43	12.9*
Subject 10	45	26	46	3.6
Subject 12	37	20	38	6.1*

Maxima of regions showing an increase in BOLD signal (*P < 0.05, significant peaks). All coordinates are in standard Montreal Neurological Institute (MNI) stereotaxic space in both the left and right hemispheres.

Additional activations within the frontal lobe were observed in the following regions (Table 1). At the group-level, a region was recruited above the horizontal ascending ramus of the lateral fissure (half) in the left hemisphere (Sprung-Much and Petrides, 2020), extending dorsally onto the antero-ventral part of the pars triangularis [MNI coordinate (*x, y, z*): −48, 32, −1, *t*-value = 5.1], where anterior area 45 lies (Petrides and Pandya, 2002). Posteriorly in the premotor region of the frontal cortex, there was increased activity in the left hemisphere at the caudalmost part of the superior frontal sulcus (sfs) at the intersection with the superior precentral sulcus (sprs) (Drudik and Petrides, 2024) [MNI coordinate (*x, y, z*): −29, −4, 65, *t*-value = 7.3], where the saccadic eye movement area lies. Ventrally in the premotor cortex, there was activity in the left hemisphere within both banks of the posterior extension of the inferior precentral sulcus (iprs-p) (Germann et al., 2005) [MNI coordinate (*x, y, z*): −48, 5, 33, *t*-value = 6.1], where the ventral premotor frontal eye field is located.

On the medial surface of the brain, significant peaks were observed anterior to the medial precentral sulcus (mprs) bilaterally [left hemisphere MNI coordinate (*x, y, z*): −3, −4, 74, *t*-value = 9.3; right hemisphere MNI coordinate (*x, y, z*): 3, −1, 72, *t*-value = 9.5], where the supplementary frontal eye field lies and within the cingulate gyrus (Petrides, 2019) [MNI coordinate (*x, y, z*): −2, 17, 33, *t*-value = 9.3] (Table 1).

### 3.2 Posterior activations

Outside the frontal lobe, group-level analysis revealed significant foci within the cortex of the left intraparietal sulcus. There were three antero-posterior foci of activity: within the depth of the sulcus of Jensen (aips-J) near the inferior termination of the superior parietal sulcus (sps) (Drudik et al., 2023; Zlatkina and Petrides, 2014) [MNI coordinate (*x, y, z*): = −30, −58, 59, *t*-value = 6.1], within the depth of the sulcus of Brissaud (sB) [MNI coordinate (*x, y, z*): = −18, −73, 51, *t*-value = 5.2], and within the paraoccipital part of the intraparietal sulcus (ips-po) (Petrides, 2019) [MNI coordinate (*x, y, z*): = −30, −85, 26, *t*-value = 4.4] (see Table 1). In addition, there was a significant peak within the posterior part of the superior parietal lobule (SPL), in the caudal sps (Drudik et al., 2023; Petrides, 2019) [MNI coordinate (*x, y, z*): = −10, −70, 59, *t*-value = 5.7]. Importantly, within the posterior ventral temporal lobe, the instructed allocation of attention to the non-spatial visual perceptual stimuli led to increased activity, bilaterally, within the posterior fusiform gyrus (Petrides, 2019), namely the cortical region that is involved in the perception of visual non-spatial stimuli (see Table 1 for the significant peaks within the fusiform gyrus, as well as other significant peaks).

## 4 Discussion

The present fMRI study provides novel evidence demonstrating, for the first time, in healthy human subjects the involvement of the posterior MFG, where area 8Av lies, when selectively allocating attention to *non-spatial* visual stimuli, i.e., stimuli that are defined by their non-spatial visual perceptual features, rather than their location in space. This demonstration is consistent with previous research on the effect of frontal lesions in patients that included the posterior prefrontal region (Petrides, 1985a; Petrides, 1990) and lesions of the periarcuate region in macaque monkeys (Petrides, 1985b; Petrides, 1987), i.e., where area 8Av is found in human and macaque brains, respectively (Petrides and Pandya, 1999). These lesions resulted in a deficit in the conditional allocation of attention to non-spatial, as well as to spatial stimuli. Note that previous functional neuroimaging in healthy adults had only demonstrated the involvement of area 8Av in the allocation of attention to locations in the visual environment (Germann and Petrides, 2020a; Germann and Petrides, 2020b) and, therefore, the question remained whether area 8Av also regulates the allocation of attention to non-spatial visual stimuli, i.e., visual perceptual stimuli independent of their location. The present fMRI study clearly demonstrated activation of area 8Av during the instructed allocation of attention to visual stimuli that were independent of their location. In addition, the present investigation demonstrated the co-activation of area 8Av with premotor frontal areas known to be involved in the control of eye movements and particular areas in the posterior parietal and posterior ventral temporal regions involved in the perceptual processing of stimuli in the environment. Thus, it revealed the essential cortical areas that comprise the functional network involved in the selective allocation of attention to non-spatial visual perceptual stimuli in the environment.

In the present fMRI study, when a comparison of functional activity was conducted between the instruction phases of the experimental and control conditions, the latter not requiring the covert allocation of attention based on instruction cues, an increase in activity was observed within the ventrocaudal MFG, where 8Av is found (see Figure 4). This functional activation was significant in the left hemisphere at both the group level (Table 1) and in individual-subject analysis which, also, revealed a corresponding increase in activity in the specific region of interest in the right hemisphere in seven of the twelve subjects (Table 2). The lateralization of the results within the left hemisphere in most subjects (see Table 1) may be understood in the context of the dominant role of the left hemisphere in language processing in the human brain (Milner et al., 1964; Petrides and Milner, 1982; Rasmussen and Milner, 1975). In particular, the verbalizable nature of the colored instruction cues (e.g., if green instruction cue, attend to visual perceptual stimulus X, but if red instruction cue, attend to visual perceptual stimulus Y) may be producing greater activation in the appropriate area 8Av and co-activated areas in the language dominant left hemisphere.

The bilateral functional activity peaks observed in the fusiform gyrus in the present fMRI study (Table 1) were of particular interest because the fusiform gyrus in the human brain is the final stage in the perceptual processing of visual stimuli (e.g., faces, abstract visual shapes, objects) in the ventral occipito-temporal stream (Allison et al., 1999; Grill-Spector, 2003; Kanwisher et al., 1997; Nasr et al., 2011; Weiner and Zilles, 2016). As is well known from studies in macaque monkeys, the processing of the perceptual features of visual stimuli involves the ventral occipito-temporal stream, in contrast to the dorsal occipito-parietal stream that is involved in the processing of the spatial aspects of visual stimuli (Mishkin and Ungerleider, 1982; Mishkin et al., 1983). Furthermore, occipito-temporal regions have been shown to be anatomically connected to area 8Av in the macaque monkey (Petrides and Pandya, 1999; Petrides and Pandya, 2002). Thus, as predicted, the present functional neuroimaging study demonstrated increased activity in the fusiform gyrus, bilaterally, when subjects were cognitively allocating attention to the associated visual perceptual aspects of the target stimulus in mind. Note that the selective attention network demonstrated in the present fMRI study involved primarily left hemisphere structures and, thus, interaction with the left hemisphere fusiform gyrus. However, given the non-spatial nature of the visual perceptual stimuli used (visual abstract designs), there was also activity in the corresponding right hemisphere fusiform gyrus (Table 1).

In the experimental condition of the present investigation, when the color instruction cue appears, the subject must actively retrieve the specific non-spatial stimulus from memorized knowledge and allocate attention to this non-spatial visual stimulus, i.e., if instruction cue A, retrieve and cognitively attend to target stimulus X, but if instruction cue B, retrieve and attend to target stimulus Y, etc. Anatomical connectivity studies in the macaque monkey have demonstrated that frontal area 8Av is strongly connected with neighboring area 45 (Petrides and Pandya, 1999; Petrides and Pandya, 2002) (see Figure 2) that is known to play a major role in the selective retrieval of relevant information from posterior cortical areas (Kostopoulos and Petrides, 2003; Kostopoulos and Petrides, 2008). Thus, in the present study, area 45, which is involved in the selective retrieval of information from memory, is co-activated with adjacent area 8Av, which is allocating attention to the retrieved stimulus (Table 1).

The covert allocation of attention to a particular environmental stimulus by area 8Av will also set in a state of readiness the premotor frontal eye field areas that would normally organize and execute eye movements to look at the selected visual target stimulus. In the lateral surface of the frontal lobe, two significant activity loci were observed that have been implicated in eye movement related activity. Increased activity was observed at the intersection of the superior precentral sulcus (sprs) with the superior frontal sulcus (sfs) (Drudik and Petrides, 2024), which is the region that has been repeatedly implicated in saccadic eye movement activity (Amiez and Petrides, 2018). In addition, there was increased activity within both banks of the posterior extension of the inferior precentral sulcus (iprs-p) (Germann et al., 2005), i.e., within the ventral premotor eye movement area (see Table 1). It has been suggested that the ventral premotor eye field may be involved in blinking responses (Kato and Miyauchi, 2003a; Kato and Miyauchi, 2003b). Furthermore, there was activity in the medial frontal lobe within the supplementary eye field in dorsal area 6 immediately anterior to the medial precentral sulcus (mprs), as well as within the cingulate/paracingulate region involved in eye movement activity (Amiez and Petrides, 2014). In the present functional neuroimaging study, we observed activity within these frontal eye field (FEF) regions, found posterior and medial to area 8Av (Petrides and Pandya, 1999), when the instruction cue was presented. We suggest that the higher-order prefrontal cortical area 8Av is involved in the executive process of allocating *cognitively* attention to a specific stimulus and communicates with the eye field regions to set them in a state of readiness to look at the object of attention as soon as it appears.

In the parietal lobe, activity peaks were observed within the intraparietal sulcal region (Zlatkina and Petrides, 2014) of the left hemisphere (Table 1), namely the parietal cortical region that is known to be involved in the organization of motor activity in space, such as eye movements (Grefkes and Fink, 2005). Tract tracing studies in macaque monkeys have demonstrated that the cortex within the intraparietal sulcus is directly connected with higher order prefrontal cytoarchitectonic areas 8Av and 45 (Petrides and Pandya, 1999; Petrides and Pandya, 2002) [see also case 3 in Petrides and Pandya (1999)]. The lateral intraparietal cortex is also connected with the FEF which in the macaque monkey lies just posterior to area 8Av within the arcuate sulcus (Huerta et al., 1987; Stanton et al., 2005). Functional imaging in human subjects has implicated this cortex as the putative lateral intraparietal area (Astafiev et al., 2003; Bremmer et al., 2001; Corbetta et al., 1998; Shikata et al., 2008) which, in the macaque monkey, has been shown to be involved in visual attention by anticipating and executing saccades (Bisley and Goldberg, 2003; Bisley et al., 2011; Kalaska, 1996; Snyder et al., 1997). Thus, the frontal activity peaks observed in the present fMRI study were co-activated with the intraparietal cortical region in the left hemisphere for the coordination of visuospatial attention to focus the eyes on a particular object in space.

In this investigation, activity was also observed within the left posterior parietal cortex, along and within the posterior intraparietal sulcus, i.e., within the cortex surrounding the sulcus of Brissaud (sB) and the paraoccipital part of the intraparietal sulcus (ips-po), as well as within the posterior SPL (Drudik et al., 2023; Petrides, 2019; Zlatkina and Petrides, 2014). Recent investigations have demonstrated that the macaque posterior parietal region involves several cortical areas, including the posterior part of the SPL that is involved in multisensory integration of certain aspects of visual and somatotopic processing. The caudal superior parietal region is connected directly with area 8Av [see case 3 in Petrides and Pandya (1999)] and the rostral premotor cortex (area 6) (Huerta et al., 1987; Stanton et al., 2005) [see case 1 in Petrides and Pandya (1999)], a region that is also reciprocally connected with area 8Av and found adjacent to the functional activity that was observed in the present investigation. Thus, this activity is a continuation of the activity observed within the intraparietal sulcal region involved in guiding the spatial focus of attention during attention shifting (Corbetta et al., 1995; Rushworth et al., 2001; Vandenberghe et al., 2001; Wager et al., 2004). Interestingly, since the present investigation examined the allocation of attention to non-spatial stimuli, we see, for the first time, the involvement of posterior parietal structures in the modulation of attentional selection to non-spatial stimuli that are embedded in space. This activity represents the state of readiness, on a particular trial, to allocate attention to the location of the visual perceptual stimulus to which attention was directed.

Several investigators have interpreted the interaction between the premotor FEF and the cortex within the intraparietal sulcus as a network establishing a “spatial priority map,” i.e., the current location of a stimulus that is the target of our attention and which will, thus, become the focus of a looking response. Note that both area 8Av (Petrides and Pandya, 1999) and the FEF (Huerta et al., 1987; Stanton et al., 2005) are connected with the intraparietal sulcus. The stimulus that is the object of our attention is thought to be held in this “spatial priority map.” Simultaneous electrophysiological recordings of single neurons in the FEF and the lateral intraparietal sulcus (LIP) of macaque monkeys demonstrated that the processing of stimulus identity and position in LIP preceded activity in FEF and that the signal within the FEF reflected the mapping of the instruction cue on the priority map that guides the focus of attention and looking response (Ibos et al., 2013). In another single neuron recording study (Sapountzis et al., 2018), the LIP activity was shown to reflect the similarity of stimuli to the target, while FEF activity integrated perceptual relevance based on oculomotor decisions. In yet another study (Zhou and Desimone, 2011), researchers recorded neural responses from the macaque FEF and visual occipital area V4, i.e., one of the later occipital stages in the processing of visual information. The data suggested that V4 provides sensory information about the visual perceptual features of the target, whereas the FEF provides a top-down attentional bias toward the target features. The above neurophysiological findings are entirely consistent with the interpretation of the present fMRI results. We argue that the activation in high-level prefrontal area 8Av cognitively allocates attention to a particular stimulus in the environment and sets in a state of readiness the posteriorly located FEF that will organize eye movements to look at the attended target. In the present fMRI experimental design, when the instruction cue is shown, the target stimuli are not in view and, thus, we demonstrate the involvement of area 8Av in the purely *cognitive* allocation of attention to the target stimuli and the simultaneous state of readiness of the frontal eye field areas to move the eyes to look at the target of our attention as soon as it appears.

In conclusion, the present study is the first to demonstrate the involvement of the posterior middle frontal gyrus, where area 8Av is found, in the high-level allocation of attention to *non-spatial* visual stimuli based on conditional instruction cues. The whole-brain analysis revealed other co-activated areas, such as the functional involvement of area 45 that is known to be involved in the selective retrieval of acquired knowledge, premotor areas involved in the organization of eye movements in interaction with posterior parietal regions where the current location of the target stimulus is coded, and the fusiform gyrus in the ventral temporal region that is involved in visual perceptual processing. Thus, these results highlighted the critical functional network involved in the cognitive allocation of attention to non-spatial stimuli in the environment.

## Data Availability

All data needed to evaluate the conclusions in the manuscript are present in the manuscript. The data that support the findings of this study are available from the corresponding author upon reasonable request.
