# Supplementary material for: The frontal and posterior cortical areas involved in the non-spatial visual allocation of attention in the human brain: a functional neuroimaging study
Source: Front Neurosci. 2025 Jan 22;18:1472114. doi: 10.3389/fnins.2024.1472114 (PMC11794536; doi:10.3389/fnins.2024.1472114)
Supplement: Supplementary file 1 [file Data_Sheet_1.docx]

Supplementary Material

# Supplementary Data

## Supplementary Figures


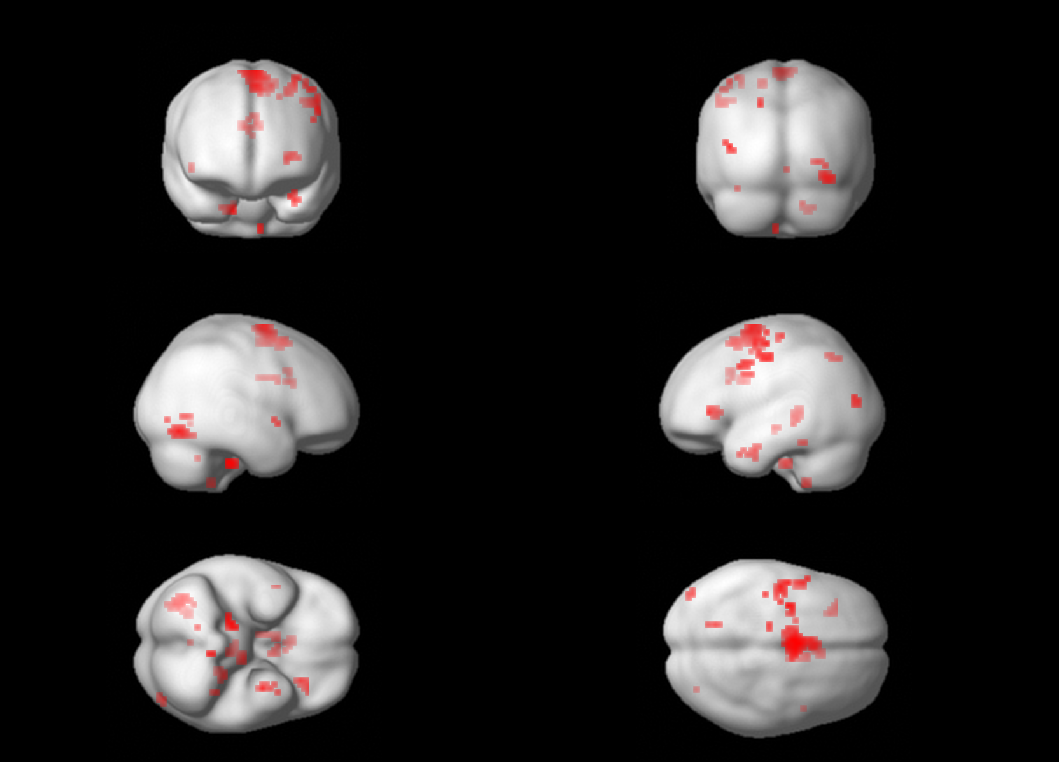


**Supplementary Figure 1.** SPM output of whole-brain cortical activity during the instruction phase of the task, overlaid on the smoothed average surface to visualize the entire cortical network co-activated with the region of interest, area 8Av. See *Discussion* section of the present manuscript for the detailed interpretation of the represented peaks. SPM outputs were thresholded at p < 0.001, uncorrected, for visualization purposes.


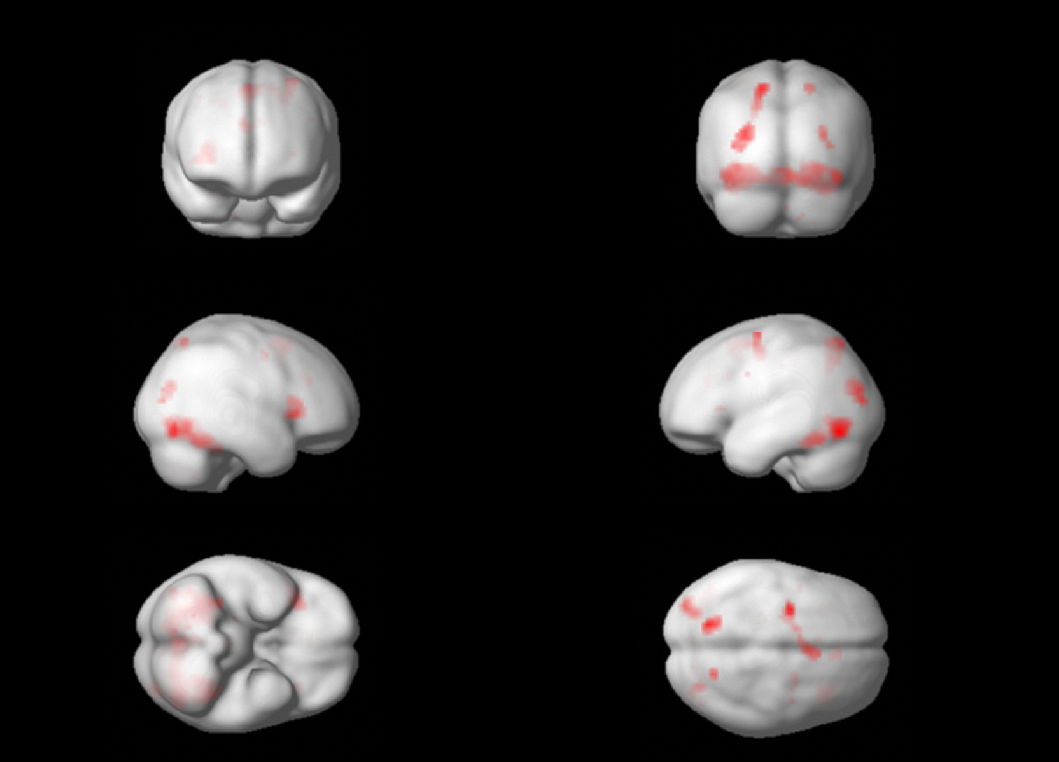


**Supplementary Figure 2.** SPM output of whole-brain cortical activity during the target phase of the task, overlaid on the smoothed average surface. SPM outputs were thresholded at p < 0.001, uncorrected, for visualization purposes.
